# Influence of high glucose in the expression of miRNAs and IGF1R signaling pathway in human myometrial explants

**DOI:** 10.1007/s00404-020-05940-5

**Published:** 2021-02-11

**Authors:** Rodolfo R. Favaro, Diana M. Morales-Prieto, Jörg Herrmann, Jürgen Sonnemann, Ekkehard Schleussner, Udo R. Markert, Telma M. T. Zorn

**Affiliations:** 1 Placenta Lab, Department of Obstetrics, Jena University Hospital, Jena, Germany; 2 Laboratory of Reproductive and Extracellular Matrix Biology, Department of Cell and Developmental Biology, Institute of Biomedical Sciences, University of São Paulo, São Paulo, Brazil; 3 Department of Gynecology and Obstetrics, Hufeland Klinikum, Weimar, Germany; 4 Department of Pediatric Hematology and Oncology, Children’s Clinic, Jena University Hospital, Jena, Germany

**Keywords:** Human, Myometrium, High glucose, IGF1R signaling pathway, miRNAs

## Abstract

**Purpose:**

Several roles are attributed to the myometrium including sperm and embryo transport, menstrual discharge, control of uterine blood flow, and labor. Although being a target of diabetes complications, the influence of high glucose on this compartment has been poorly investigated. Both miRNAs and IGF1R are associated with diabetic complications in different tissues. Herein, we examined the effects of high glucose on the expression of miRNAs and IGF1R signaling pathway in the human myometrium.

**Methods:**

Human myometrial explants were cultivated for 48 h under either high or low glucose conditions. Thereafter, the conditioned medium was collected for biochemical analyses and the myometrial samples were processed for histological examination as well as miRNA and mRNA expression profiling by qPCR.

**Results:**

Myometrial structure and morphology were well preserved after 48 h of cultivation in both high and low glucose conditions. Levels of lactate, creatinine, LDH and estrogen in the supernatant were similar between groups. An explorative screening by qPCR arrays revealed that 6 out of 754 investigated miRNAs were differentially expressed in the high glucose group. Data validation by single qPCR assays confirmed diminished expression of miR-215-5p and miR-296-5p, and also revealed reduced miR-497-3p levels. Accordingly, mRNA levels of *IGF1R* and its downstream mediators *FOXO3* and *PDCD4,* which are potentially targeted by miR-497-3p, were elevated under high glucose conditions. In contrast, mRNA expression of *IGF1*, *PTEN, and GLUT1* was unchanged.

**Conclusions:**

The human myometrium responds to short-term exposure (48 h) to high glucose concentrations by regulating the expression of miRNAs, *IGF1R* and its downstream targets.

**Electronic supplementary material:**

The online version of this article (10.1007/s00404-020-05940-5) contains supplementary material, which is available to authorized users.

## Introduction

The myometrium comprises the contractile unit of the uterus, in which bundles of smooth muscle cells are organized in two distinct layers supported by connective tissue in-between [1]. In addition to its prominent role during labor, the myometrium also contributes to menstrual discharge [2], sperm and embryo transport [3] and uterine blood flow regulation [4]. Therefore, myometrial alterations are associated with reproductive disorders [5]. This is especially relevant in the context of diabetes, where both preterm deliveries and cesarean rates are considerably higher due to labor dysfunctions [6–8]. The rise in diabetes incidence [9] reinforces the need for a better comprehension regarding the influence of this disease upon the myometrium in order to prevent or treat its adverse consequences.

While insulin deficiency caused by beta-cell destruction characterizes type 1 diabetes, type 2 diabetes is promoted by impairment of insulin actions due to alterations in insulin receptor signaling, a phenomenon known as insulin resistance. Gestational diabetes mellitus, a form of diabetes that arises during pregnancy, shares similar features of type 2 diabetes. In either case, glucose accumulates in the bloodstream. Hyperglycemia and associated metabolic disarrangements are detrimental to cells leading to tissue and organ dysfunctions [10].

Myometrial contractility has been shown to be impaired in diabetic women [6, 11] and animal models of diabetes [12–14]. Results from our group in type 1 diabetic mice demonstrate the profound effects of this disease on the pregnant myometrium. Reduction of smooth muscle cell proliferation was associated with reduced thickness of the muscle layers. Moreover, alterations on the contractile apparatus of smooth muscle cells and deposition of collagens and proteoglycans as well as disorganization of collagen fiber orientation were reported [15, 16]. Decreased expression of calcium channels and intracellular calcium levels together with reduced muscular mass have been linked to poor myometrial contractility and labor deficiency in diabetic women [6].

Complications promoted by diabetes in susceptible tissues are associated with changes in the expression of hormones, cytokines and growth factors. Insulin-like growth factor-1 receptor (IGF1R) signaling pathway regulates several cellular responses in physiological and pathological conditions, including cell proliferation, differentiation and survival [17, 18]. Alterations in this pathway have been demonstrated to be present in different organs affected by diabetes [19]. However, the effects of high glucose on human myometrium and the association with IGF1R pathway remain to be investigated.

Binding of insulin-like growth factor-1 (IGF1) to IGF1R results in autophosphorylation and activation of this receptor followed by phosphorylation of cellular substrates. IGF1R signals through different signaling cascades, including mitogen-activated protein kinases (MAPKs) and phosphatidylinositol 3-kinase (PI3K)/AKT. Activation of PI3K phosphorylates the lipid mediator phosphatidylinositol-4,5-bisphosphate (PIP2) to phosphatidylinositol-3,4,5-trisphosphate (PIP3) [17, 18]. One of the major actions of PIP3 involves the activation of AKT, which in turn has several downstream effectors, including forkhead box class O3 (FOXO3) transcription factor and programmed cell death protein 4 (PDCD4). FOXO3 is associated with cell metabolism, survival, and inflammatory processes [20], whereas PDCD4 is a tumor suppressor protein that regulates transcription, translation, DNA-damage response, cell death, and motility [21]. Phosphatase and tensin homolog (PTEN) counteracts the activity of PI3K. This enzyme dephosphorylates PIP3, converting it back into PIP2, and thus downregulates PI3K signaling and AKT activation [17, 18].

Several regulatory mechanisms acting at both transcriptional and post-transcriptional levels adjust gene expression on cells. Knowledge about non-coding RNA species and their relevant biological functions have significantly expanded in recent years. Amongst them, miRNAs have been reported to perform major roles in post-transcriptional regulation of mRNA levels. miRNAs are molecules of ≈ 22 nucleotides able to recognize complementary or partially complementary sequences on the 3′ untranslated region of their mRNA targets. Usually, this interaction results in transcriptional repression and increased degradation of mRNA targets [22]. The ability of miRNAs to control mRNA stability and translation confers to them a remarkable role in the regulation of cellular processes in physiological as well as in pathological conditions [23–25].

In the present study, the effects of high glucose on the expression of miRNAs and their putative mRNA targets within the IGF1R signaling pathway have been investigated in human myometrial explants.

## Methods

### Human myometrial explant culture

Human myometrial samples were collected from ten patients undergoing hysterectomy for benign reasons at the Department of Obstetrics of the Hufeland Klinikum, Weimar, Germany. All samples were derived from perimenopausal German patients with an average age of 45.9 ± 2.2 years and without hormonal treatment at the time of surgery.

The explant cultures were initiated less than 30 min after surgery. Each sample of approximately 20 mg was split in two parts and fragmented into pieces of around 2 mm^3^. Explants were cultivated for 48 h in 4 mL medium DMEM (Gibco) containing either low (5.5 mmol/L) or high glucose (25 mmol/L), 10% FCS (Product No. F7524; Sigma, Germany), penicillin and streptomycin (Gibco), under standard culture conditions (37 °C, 5% CO_2_, humidified atmosphere). After the experimental period, supernatants were collected for biochemical evaluations and the myometrial fragments processed for qPCR and histology.

### Biochemical evaluation

Supernatants collected after 48 h of cultivation were tested for glucose, lactate, LDH, and creatinine via spectrophotometry. Estrogen and progesterone levels were evaluated by electrochemiluminescence immunoassay “ECLIA” on a Roche-Cobas instrument (Roche), following manufacturer’s instructions. Values obtained on supernatants were normalized to the amount of tissue present in each well.

### Histological processing

For histological analysis, myometrial samples were fixed in Methacarn (methanol, chloroform and glacial acetic acid, 6:3:1) for 3 h and routinely processed for paraffin embedding and hematoxylin–eosin (HE) staining.

### miRNA expression profiling

After collection, myometrial explants were stored in RNAlater at − 80 °C. Subsequently, they were homogenized using a GentleMacs homogenizer with M tubes and total RNA was isolated using Trizol (Invitrogen). RNA concentration and purity were analyzed with a Qiaexpert (Qiagen) instrument. miRNA expression profile was assessed in myometrial explants from two patients cultivated in high and low glucose using TaqMan miRNA arrays A (version 2) and B (version 3) (Thermo, containing together 754 miRNAs. Data were analyzed with DataAssist v3.01 (Thermo Fisher). *RNU44*, *RNU48* and *U6* were evaluated as reference genes, with the latter selected for the analyses. Expression of *U6* and *RNU44* were also verified by qPCR using single Taqman miRNA assays. miRNAs found to be differentially expressed in the TaqMan miRNA arrays were validated with single TaqMan miRNA assays using myometrial explants from ten patients. Data are presented as 2^−∆Ct^ ± standard error of mean (SEM). Experiments with TaqMan miRNA arrays and TaqMan single assays were carried out following manufacturer’s protocols (Thermo Fisher) and a previous publication from our group [26]. The list of miRNA Assay IDs can be found in Table 1 from the Online Resource 1. The complete data set of the TaqMan miRNA arrays A and B are present in the Online Resource 2.

### miRNA target prediction in silico

TargetScan (v7.2; targetscan.org) and DIANA-microT-CDS (v5.0 microrna.gr/microT-CDS) databases were used to identify potential targets of miRNAs using standard configurations.

### Gene expression analysis

One µg of total RNA was used for cDNA conversion using the High Capacity RNA to cDNA Conversion Kit (Thermo Fisher, USA). Following, mRNA expression of Glucose transporter 1 *(GLUT1/SLC2A1)*, *IGF1*, *IGF1R*, *PTEN*, *FOXO3*, and *PDCD4* was examined by TaqMan assays. Glycerinaldehyd-3-phosphat-Dehydrogenase *(GAPDH)*, Actin Beta *(ACTB)* and Peptidylprolyl isomerase A *(PPIA)* have been tested and the latter was selected as reference gene. The respective mRNA TaqMan assay IDs can be found in Table 2 from the Online Resource 1. Data are presented as 2^−∆Ct^ ± SEM.

### Statistical analysis

Comparisons between the two experimental groups (high vs. low glucose) were run in GraphPad software. Since each myometrial sample was split into two parts and cultivated in either high or low glucose conditions, the paired Student’s t test was applied. Values of *p* < 0.05 were considered statistically significant.

## Results

### Biochemical parameters

After 48 h of cultivation, glucose levels were still markedly higher in the high glucose group than in the low glucose group (29.2 ± 0.7 vs. 6.5 ± 0.2 mmol/L; *p* < 0.001). Lactate (0.26 ± 0.03 vs. 0.24 ± 0.02 µmol/L; *p* = 0.2229), creatinine (0.91 ± 0.8 vs. 1.06 ± 0.11 µmol/L; *p* = 0.1948), LDH (0.020 ± 0.004 vs. 0.018 ± 0.002 µmol/L; *p* = 0.3077) and estrogen levels (2.52 ± 0.17 vs. 2.26 ± 0.16 µmol/L; *p* = 0.0582) were comparable between low and high glucose conditions, respectively. Progesterone was not detected in the supernatant of either group.

### Histological evaluation

**Fig. 1:**
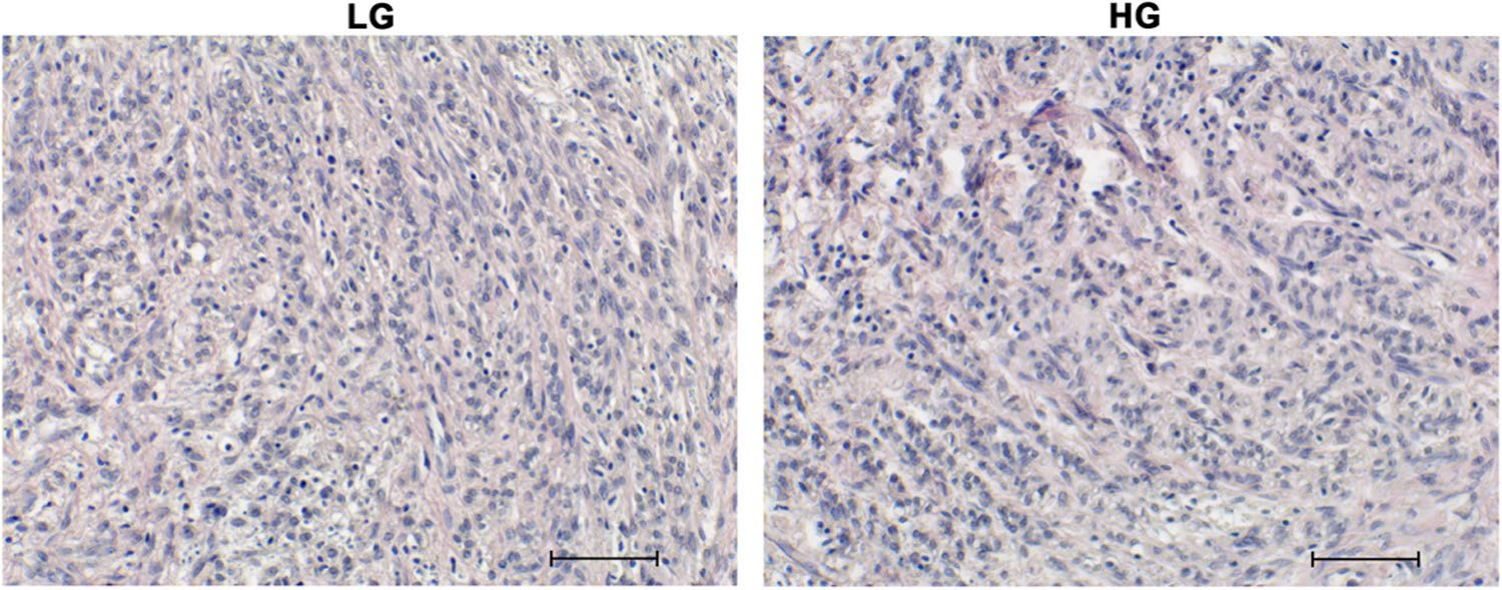
Representative histological evaluation of human myometrial explants cultured under low (LG) and high glucose (HG) conditions for 48 h. Most cell nuclei are heterochromatic, indicating the viability and transcriptional activity of the explants from both groups. Scale bar: 100 µm

Myometrial explants cultivated under high and low glucose conditions showed similar morphological aspects. No gross signals of degeneration were observed by evaluating HE-stained samples (Fig. 1).

### miRNA profiling expression and target analysis

**Fig. 2:**
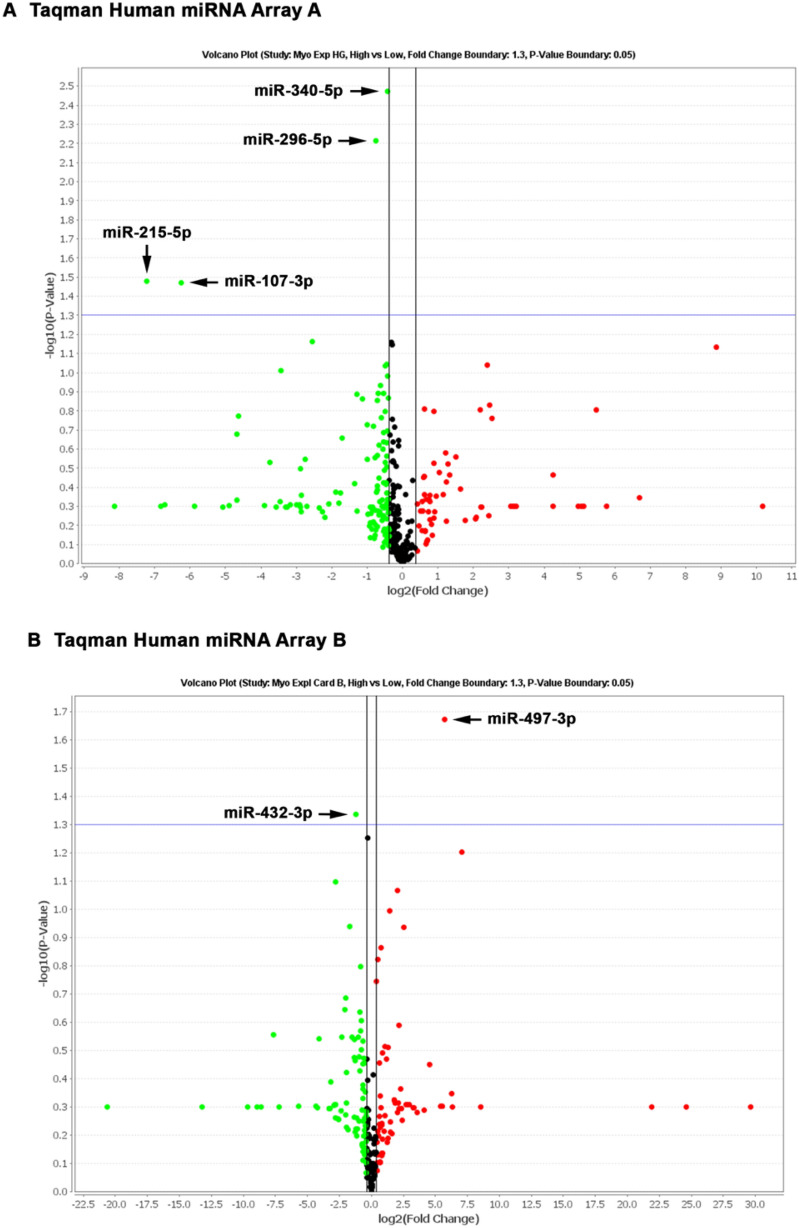
Volcano plot of miRNA expression from TaqMan miRNA array A (**a**) and B (**b**) in human myometrial explants cultured in low and high glucose for 48 h. U6 was used as reference gene. Downregulated miRNAs are shown in green and up-regulated miRNAs in red above the fold change boundary of 1.3 and p values of < 0.05 (*n* = 2)

**Fig. 3:**
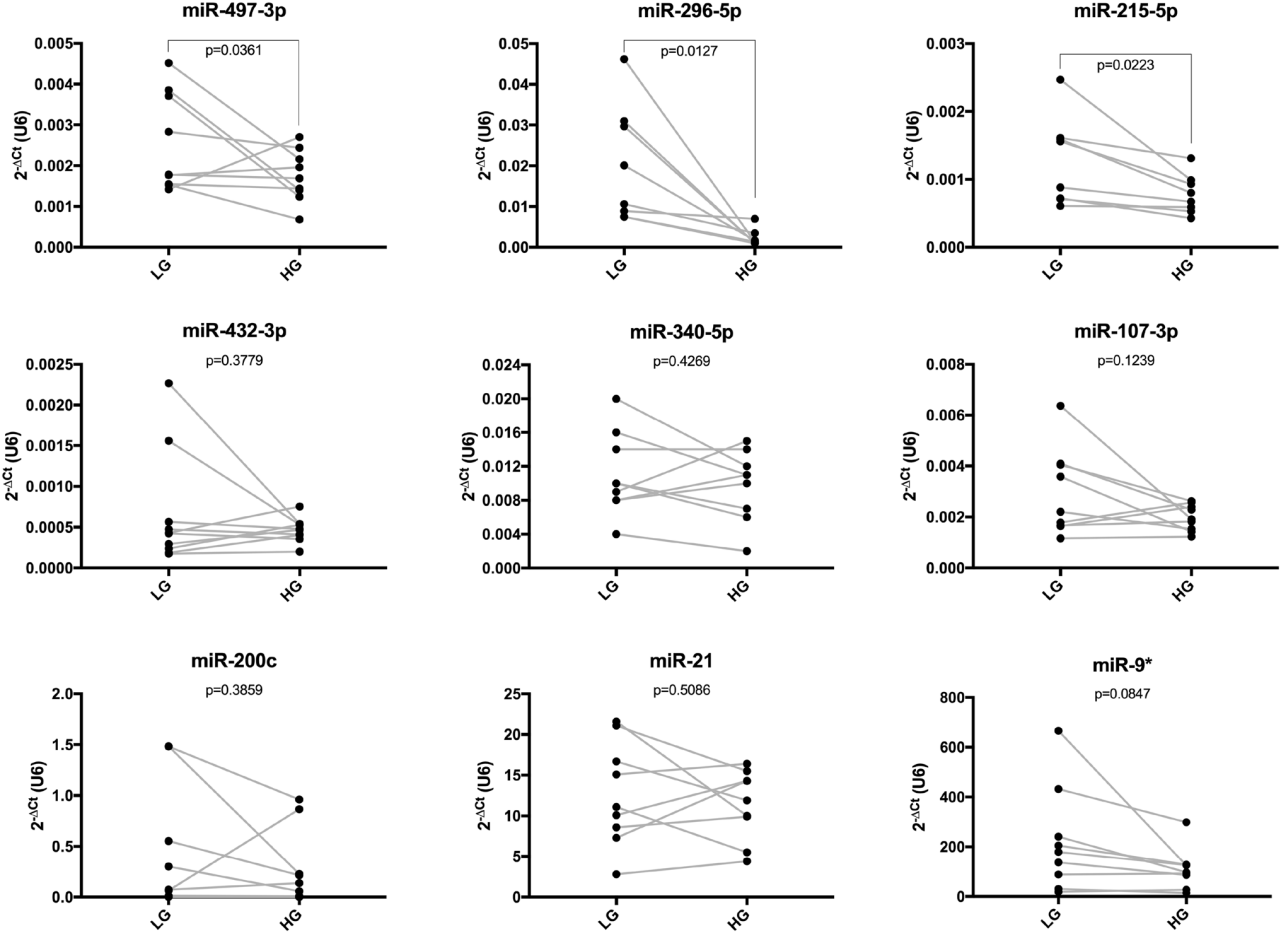
Analysis of miR-497-3p, miR-296-5p, miR-215-5p, miR-432-3p, miR-340-5p, miR-107-3p, miR-200c-3p, miR-21-5p, miR-9-3p expression by qPCR in human myometrial explants cultured in low (LG) and high glucose (HG) for 48 h. Observe the decreased expression of miR-215-5p, miR-296-5p and miR-497-3p. Gray lines connect high and low glucose dyads (*n* = 10). Data are presented as 2^−∆Ct^ to U6. Statistical differences are analyzed by the paired Student’s t test

*U6* was slightly more stable than *RNU44* (data not shown) and thus selected as reference gene for the normalization of data from qPCR arrays as well as from single qPCR assays. Values were considered significant above a fold change of 1.3 and *p* values of < 0.05. From 754 miRNAs screened with TaqMan miRNA arrays A + B, six miRNAs were found to be differentially expressed in the myometrial explants cultivated in high glucose conditions. The levels of miR-107-3p, miR-215-5p, miR-296-5p, miR-340-5p, and miR-432-3p were decreased, whereas miR-497-3p was increased (Fig. 2). Following, data from qPCR arrays were validated by single TaqMan assays in the same samples. In addition to six altered miRNAs, three other miRNAs whose levels were not changed by high glucose were also included in the analysis. The values detected by single qPCR assays were equivalent to those from qPCR arrays for all nine miRNAs evaluated, demonstrating the robustness of the approach used. When a larger number of samples (*n* = 10) was investigated, we confirmed that miR-215-5p and miR-296-5p were decreased in high glucose conditions. However, we observed a significant decrease in miR-497-3p levels (Fig. 3). This discrepancy was due to the sample included in the analysis by qPCR array, which was the only one to present increased expression of this miRNA. The differences were not statistically significant for miR-107-3p, miR-340-5p, and miR-432-3p. Moreover, in line with the miRNA arrays, expression of miR-9-3p, miR-21-5p and, miR-200c-3p was unchanged between the groups (Fig. 3).

Bioinformatical analyses using two different databases, TargetScan and DIANA-microT-CDS, predicted *IGF1* and *IGF1R* as targets of miR-497-3p. TargetScan also indicated *PTEN*, *FOXO3* and *PDCD4* as putative targets of this miRNA. Subsequently, we investigated the mRNA expression of these genes by qPCR.

### Gene expression of IGF1R signaling pathway

**Fig. 4:**
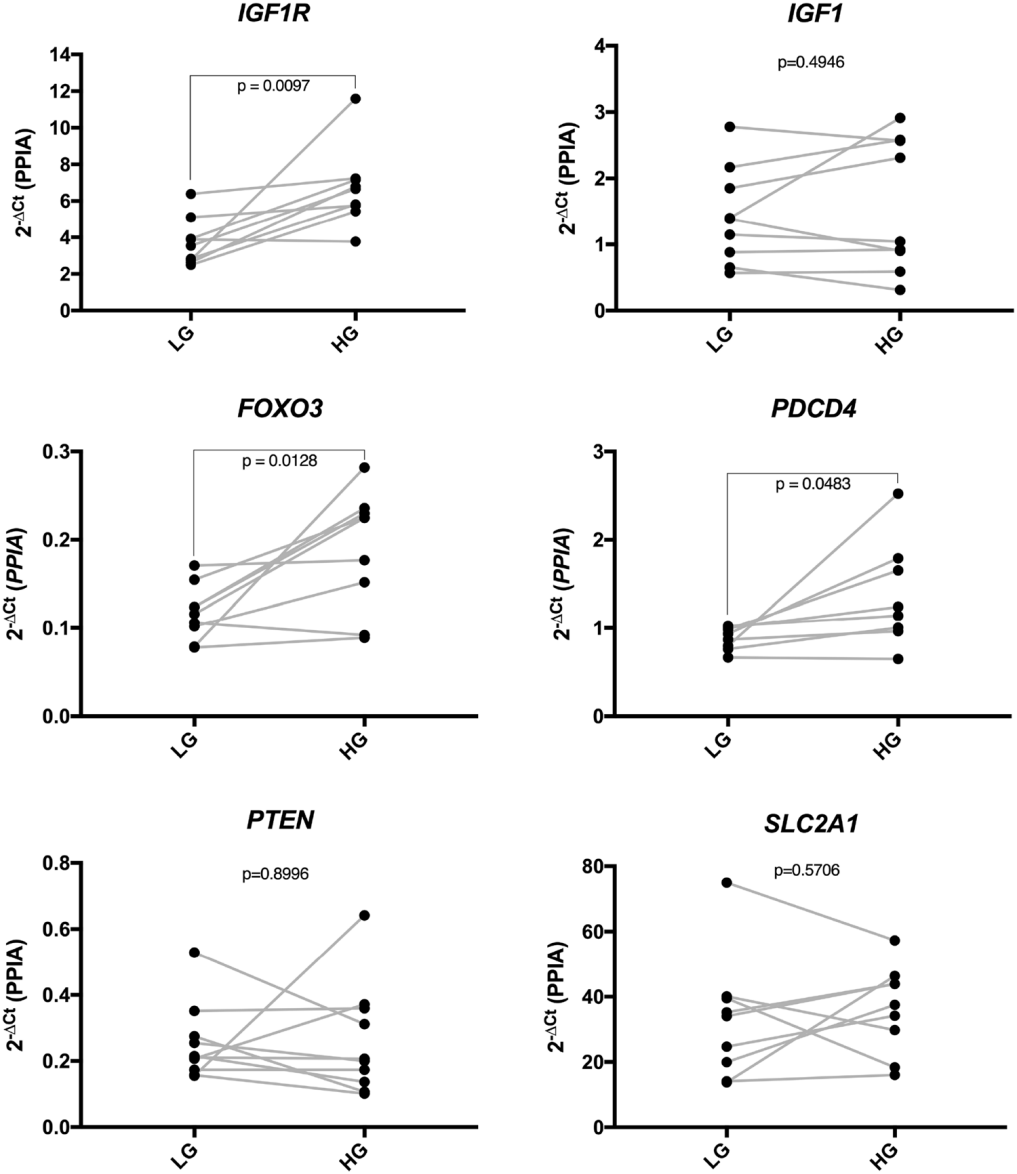
Analysis of *IGF1R, IGF1, FOXO3, PDCD4, PTEN,* and *SLC2A1* expression by qPCR in human myometrial explants cultivated in low (LG) and high glucose (HG) for 48 h. Note that high glucose increases the mRNA expression of *IGF1R*, *PTEN* and *PDCD4*. Gray lines connect high and low glucose dyads (*n* = 10). Data is presented as 2^−∆Ct^ to PPIA. Statistical differences are analyzed by the paired Student’s *t* test

*GAPDH* was not suitable as reference gene in this model due to a high variability between treatments (data not shown). *ACTB* and *PPIA* were similarly stable (data not shown), and *PPIA* has been selected for further calculations of the qPCR data. While mRNA expression of miR-497-3p targets *IGF1R*, *FOXO3,* and *PDCD4* was elevated, that of *IGF1*, *PTEN* and *SLC2A1* was not changed in the myometrial explants cultivated under high glucose conditions, as compared to low glucose (Fig. 4).

## Discussion

Despite the importance of the myometrium for reproduction and the impairments promoted by diabetes on it, surprisingly little is known on how high glucose affects this compartment. Using human myometrial explants, we demonstrate that the myometrium is sensitive to short-term exposure (48 h) of high glucose concentration. Alterations in the expression of miRNAs, *IGF1R* and its downstream mediators, *FOXO3* and *PDCD4*, have been reported. Considering the role of these molecules to myometrial biology, our results provide initial experimental evidence to clarify the pathogenic mechanisms underlying clinical manifestations of diabetic complications in the human myometrium.

As advantages of our experimental setting, we highlight the controlled culture conditions and the simultaneous comparison of samples from the same patients in both high and low glucose media. Our results show that high glucose is able to affect myometrial miRNA and mRNA expression in the absence of other endocrine-metabolic factors present in vivo. Due to limited tissue viability in culture, this study focused on short-term effects of high glucose on the human myometrium. A single time-point (48 h) and high glucose concentration (25 mmol/L) commonly employed in in vitro experiments, compared to low glucose (5.5 mmol/L), were investigated. In this context, other potential effects associated with long-lasting exposure to high glucose could not be addressed in explant cultures and require other experimental approaches. An additional limitation concerns the relatively small number of myometrial samples (*n* = 10) used in our study. Nevertheless, it was sufficient to demonstrate significant differences in miRNA and mRNA expression of myometrial explants cultivated in high glucose.

The myometrial miRNA expression profile has been investigated in different models and conditions. Several miRNAs were found to be differentially expressed by quiescent vs. in labor myometrium [27], normal myometrium vs. leiomyoma [28], during preterm labor [29] and after treatment with oxytocin [30]. Our study also shows that high glucose dysregulates myometrial miRNA expression and identifies some targets of this response. These data indicate potential targets for clinical and pharmacological intervention to prevent the deleterious effects of diabetes and high glucose in the myometrium.

A molecular screening revealed that 6 out of 754 miRNAs investigated in the myometrial explants were altered by cultivation in high glucose. Decreased expression of miR-107-5p, miR-215-5p, miR-296-5p, miR-340-5p and miR-432-3p and increased expression of miR-497-3p was observed. Validation of this data using single qPCR assays in a higher number of samples confirmed decreased expression of miR-215-5p and miR-296-5p. Although the qPCR results from samples used for the qPCR arrays were equivalent, analysis of additional samples resulted in a significant decreasing of miR-497-3p levels by high glucose.

Downregulation of miR-296-5p was also reported in the mouse pancreatic beta-cell line MIN6 cultured under high glucose concentrations [31]. Conversely, miR-215-5p was shown to be up-regulated by high glucose in mouse mesangial cells as well as in the kidney of diabetic mice [32]. To our knowledge, miR-215-5p, miR-296-5p, and miR497-3p have not been previously associated with diabetes or high glucose in humans. More data are needed to ascertain if our observations are restricted to the myometrium or if these miRNAs are also modulated by high glucose in other tissues.

IGF1, IGF1R, and IGFBPs 1–4 are expressed by the human myometrium [33]. Treatment of myometrial smooth muscle cells with IGF1 in combination with EGF and PDGF-BB stimulates their proliferation [33]. Similarly, overexpression of IGF1 in smooth muscle cells of mice promotes myometrial hyperplasia and longitudinal growth of the uterine horns [34], whereas IGF1 ablation leads to myometrial hypoplasia [35]. Shynlova et al. demonstrated in rats that the myometrium has four major phases of adaptation during pregnancy, which are associated with specific patterns of expression of IGF family members [36]. These studies highlight the roles played by IGF1 signaling on myometrium physiology.

Diabetes affects IGF1/IGF1R expression and signaling in several organs. For instance, reduced levels of IGF1R were described in the placenta of diabetic women [37]. In the human retina, diabetes decreases IGF1 expression and promotes a slight increase in IGF1R [38]. In our study, elevated expression of IGF1R without changes in IGF1 was detected in the myometrial explants cultivated under high glucose conditions. Collectively, these results show that the effects of diabetes and high glucose on IGF1/IGF1R expression are tissue-specific. Furthermore, changes observed in the expression of IGF1R in myometrial explants cultivated in high glucose may impair myometrial functioning. The applicability of IGF1R inhibitors to restore myometrial homeostasis in diabetic conditions warrant further investigation.

Target prediction with TargetScan and DIANA-microT-CDS databases predicted *IGF1R*, *IGF1, PTEN*, *FOXO3* and *PDCD4* as targets of miR-497-3p demonstrating its potential role in regulating IGF1R pathway. In accordance with decreased miR-497-3p expression, we found raised levels of *IGF1R* and of its targets *FOXO3* and *PDCD4* in the myometrial explants exposed to high glucose. Silencing of IGF1R in human non-small cell lung cancer A549 cells led to downregulation of 59 miRNAs and upregulation of 13 miRNAs, including miR-497-3p [39], indicating a regulatory reciprocity between IGF1R and miR-497-3p. Thus, we suggest that elevated expression of IGF1R and downregulation of miR-497-3p may be intrinsically associated in the myometrial explants cultivated under high glucose conditions.

Changes in the expression of miR-296-5p were described during differentiation of endothelial cells from human embryonic stem cells and human induced pluripotent stem cells [40], in preeclamptic placentas [41], in liver of nonalcoholic steatohepatitis [42], and in cancer cells. miR-296-5p acts as a tumor suppressor miRNA in breast [43], prostate [44], and non-small cell lung cancers [45], whereas in gastric cancer, this miRNA operate as an onco-miR, stimulating cell proliferation [46]. Similarly, miR-215-5p has tumor-suppressive properties in colorectal cancer, where its level is reduced [47] and tumor-promoting effects in glioma cells through elevated proliferation and reduced apoptosis [48]. miR-215-5p and miR-192-5p regulate glycolysis in colon cancer cells through Sushi Repeat Containing Protein X-Linked 2 (SRPX2) expression, and downregulation of these miRNAs is promoted by PI3K-AKT pathway [49]. PI3K-AKT has been shown to be up-regulated by high glucose in endometrial [50] and smooth muscle cells [51]. Considering the augmented expression of IGF1R, which signals through PI3K-AKT, as well as of FOXO3 and PDCD4, downstream genes of this pathway, reduced levels of miR-215-5p in the myometrial explants may be promoted via the stimulation of PI3K-AKT by high glucose. Modulation of AKT pathway may constitute a therapeutic approach to mitigate the impact of high glucose in the myometrium.

Diabetes and high glucose are associated with elevated incidence of several types of cancers [52]. Uterine leiomyomas or fibroids are benign neoplasms that arise from myometrial smooth muscle cells. Alterations on IGF1/IGF1R signaling have been reported in this condition [53]. Furthermore, both FOXO3 [54] and PDCD4 [55] are increased in leiomyoma compared with myometrial tissue. Our results show that high glucose promotes alterations in the myometrium that are also present in leiomyoma, leading to the speculation that diabetes and hyperglycemia may contribute to its development. On one hand, there is evidence showing that diabetes protects against the development of leiomyomas [56]. On the other hand, high dietary glycemic index and glycemic load are associated with elevated risk for the development of uterine leiomyoma [57]. Additional studies are required to address the influence of diabetes and high glucose to leiomyoma initiation and progression.

In conclusion, we observed that high glucose downregulated the expression of miR-215-5p, miR-296-5p and miR-497-3p in human myometrial explants. Accordingly, expression of miR-497-3p-associated targets, including IGF1R and its downstream mediators FOXO3 and PDCD4 were elevated. The implications of these findings for myometrial functionality should be evaluated in further experimental settings. Finally, our results show that short-term exposure to high glucose alters myometrial biology, reinforcing the importance of tight glycemic control in diabetic patients to prevent potential complications in this compartment.

## Electronic supplementary material

Below is the link to the electronic supplementary material.

Supplementary material 1 (DOCX 10 kb)

Supplementary material 2 (XLS 10 kb)
