# Supplementary material for: Influence of high glucose in the expression of miRNAs and IGF1R signaling pathway in human myometrial explants
Source: Arch Gynecol Obstet. 2021 Feb 11;303(6):1513–22. doi: 10.1007/s00404-020-05940-5 (PMC8087607; doi:10.1007/s00404-020-05940-5)
Supplement: Supplementary file 1 — Supplementary material 1 (DOCX 10 kb) [file 404_2020_5940_MOESM1_ESM.docx]

**Online Resource 1**

Favaro et al. Influence of high glucose in the expression of miRNAs and IGF1R signaling pathway in human myometrial explants. Arch Gynecol Obstet.

**Table 1:** Taqman assays used for the analysis of miRNA expression by qPCR (Thermo Fisher Scientific, USA)

| **miRNA** | **Assay ID** |
| --- | --- |
| hsa-miR-9-3p | 002231 |
| hsa-miR-21-5p | 000397 |
| hsa-miR-107-5p | 000443 |
| hsa-miR-200c-3p | 002300 |
| hsa-miR-215-5p | 000518 |
| hsa-miR-296-5p | 000527 |
| hsa-miR-340-5p | 002258 |
| hsa-miR-432-3p | 001027 |
| hsa-miR-497-3p | 002368 |
| RNU44 | 001094 |
| U6 snRNA | 001973 |

**Table 2:** Taqman assays used for the analysis of gene expression by qPCR (Thermo Fisher Scientific, USA)

| **Gene** | **Assay ID** |
| --- | --- |
| *ACTB* | Hs01060665_g1 |
| *FOXO3* | Hs00818121_m1 |
| *GAPDH* | Hs02758991_g1 |
| *IGF1* | Hs01547656_m1 |
| *IGF1R* | Hs00609566_m1 |
| *PDCD4* | Hs00377253_m1 |
| *PPIA* | Hs04194521_s1 |
| *PTEN* | Hs02621230_s1 |
| *SLC2A1* | Hs00892681_m1 |
